# Translating tumor biology into personalized treatment planning: analytical performance characteristics of the Onco*type *DX^® ^Colon Cancer Assay

**DOI:** 10.1186/1471-2407-10-691

**Published:** 2010-12-23

**Authors:** Kim M Clark-Langone, Chithra Sangli, Jayadevi Krishnakumar, Drew Watson

**Affiliations:** 1 Genomic Health, Inc., Redwood City, CA, 94063, USA; 2 St. Jude Medical, Sunnyvale, CA, 94086, USA

## Abstract

**Background:**

The Onco*type *DX^® ^Colon Cancer Assay is a new diagnostic test for determining the likelihood of recurrence in stage II colon cancer patients after surgical resection using fixed paraffin embedded (FPE) primary colon tumor tissue. Like the Onco*type *DX Breast Cancer Assay, this is a high complexity, multi-analyte, reverse transcription (RT) polymerase chain reaction (PCR) assay that measures the expression levels of specific cancer-related genes. By capturing the biology underlying each patient's tumor, the Onco*type *DX Colon Cancer Assay provides a Recurrence Score (RS) that reflects an individualized risk of disease recurrence. Here we describe its analytical performance using pre-determined performance criteria, which is a critical component of molecular diagnostic test validation.

**Results:**

All analytical measurements met pre-specified performance criteria. PCR amplification efficiency for all 12 assays was high, ranging from 96% to 107%, while linearity was demonstrated over an 11 log_2 _concentration range for all assays. Based on estimated components of variance for FPE RNA pools, analytical reproducibility and precision demonstrated low SDs for individual genes (0.16 to 0.32 C_T_s), gene groups (≤0.05 normalized/aggregate C_T_s) and RS (≤1.38 RS units).

**Conclusions:**

Analytical performance characteristics shown here for both individual genes and gene groups in the Onco*type *DX Colon Cancer Assay demonstrate consistent translation of specific biology of individual tumors into clinically useful diagnostic information. The results of these studies illustrate how the analytical capability of the Onco*type *DX Colon Cancer Assay has enabled clinical validation of a test to determine individualized recurrence risk after colon cancer surgery.

## Background

The emergence of personalized medicine as a central healthcare theme has led to an explosion of research dedicated to finding molecular markers for prediction of disease outcome and treatment benefit. However, achieving clinical validation for molecular tests has proven to be challenging given the dual needs for selection of high quality molecular markers and the provision of an analytically sound test process for their measurement. Indeed, the clinical utility of molecular biomarkers may be limited unless they are provided in the context of a robust, well-characterized diagnostic test with documented accuracy, precision, and reproducibility. The Onco*type *DX Breast Cancer Assay has met this standard as an analytically and clinically validated high complexity, multi-analyte, RT-PCR test to predict the likelihood of recurrence and response to chemotherapy in estrogen receptor-positive, node-negative and node-positive breast cancer patients [1,2]. With over 160,000 tests performed since its launch in 2004, and its inclusion in the National Comprehensive Cancer Network (NCCN) and American Society Clinical Oncology (ASCO) clinical practice guidelines, the Onco*type *DX Breast Cancer Assay has become standard of care for individualized treatment decision-making in early stage breast cancer [3-5].

For patients diagnosed with stage II colon cancer, the decision of whether or not to receive adjuvant chemotherapy has been a significant challenge, in part due to limitations in existing methods for assessing recurrence risk. Although pathologic T4 stage and mismatch repair deficiency (MMR-D) can identify patients at significantly higher or lower recurrence risk, respectively [6,7], these two markers account for only 25-30% of stage II patients. For the majority of stage II colon cancer patients, who would be characterized as having standard risk, there has been a major need for better prediction of individual recurrence risk [6]. Based on the results of the clinical validation study of the 12 gene Onco*type *DX Colon Cancer Assay [7], stage II colon cancer patients (particularly those with T3 disease and mismatch repair proficient (MMR-P) tumors) are now empowered to make more informed treatment decisions with quantitative, individualized recurrence risk information based on the molecular profile of their tumor.

**Figure 1:**
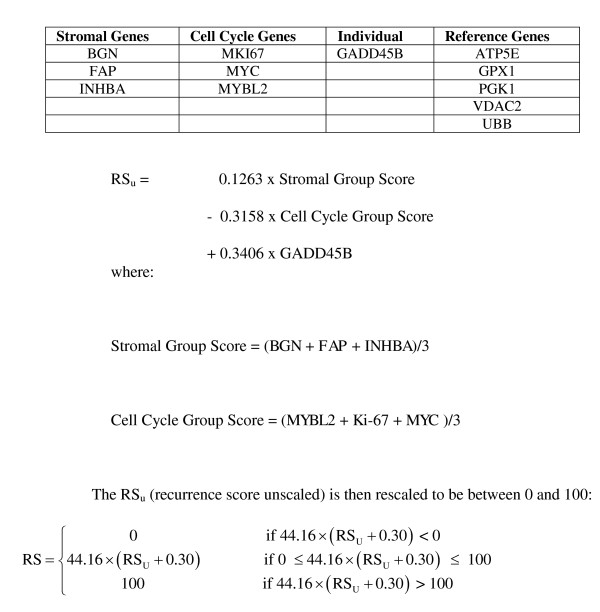
**RS algorithm**. Shown are gene names, their associated gene group and the algorithm for calculation of the Recurrence Score.

The seven cancer-related genes in the Onco*type *DX Colon assay were selected from a panel of 761 genes for their consistent association with colon cancer recurrence in 4 large and independent development studies [6]. Three of these genes are associated with activated stroma (*BGN, INHBA *and *FAP*), three represent a cell cycle pathway (*MK167, MYBL2 *and *MYC*) and one can be characterized as part of an early-response or genotoxic stress pathway (*GADD45B*). For each patient, expression values from the co-expressed stromal genes are aggregated to form a stromal gene score (SGS) and the expression values of the three cell cycle genes are aggregated to form a cell cycle gene score (CCGS). The SGS, CCGS and the expression of *GADD45B *are combined to generate the final RS which provides an individualized risk estimate for colon cancer recurrence (see Figure 1).

The expression levels of these genes may vary both within and between FPE blocks from the same patient's tumor. However, all sources of variability are inherently incorporated into the clinical validation of the Onco*type *DX Colon Cancer Assay, and therefore embedded within the estimated risk of disease recurrence obtained for each RS value. However, one source of variability which can be controlled in a molecular diagnostic test is that derived from the assay process (analytical variability). In fact, it is this source of variability which has to be controlled if a consistent and reliable test result is to be provided to patients on a daily basis. Prior to performing the clinical validation study of the 12 gene RS assay we established pre-determined assay performance criteria and performed analytical validation studies, designed to demonstrate that the process was well-controlled and capable of reproducibly translating tumor biology into clinically actionable information. We report here the design and results of the suite of analytical validation studies that characterize the performance of the assay.

## Methods

### Gene selection

The seven cancer-related genes (*BGN, MYC, FAP, GADD45B, INHBA, MK167 *and *MYBL2*) and five reference normalization genes (*ATP5E, GPX1, PGK1, VDAC2 *and *UBB*) were selected from 761 genes, based on the results of four development studies which included over 1800 patients [6].

### Tumor blocks and samples

FPE colon cancer blocks (Stage II adenocarcinoma and mucinous carcinoma) from recent surgeries (within 3-6 months of testing) were obtained under an Institutional Review Board approved protocol. The selected blocks were representative of the tumor block type expected to be received in the clinical reference laboratory (with respect to tumor type, fixative and age). Samples with no tumor or very little tumor (< 5% tumor present) were excluded. In studies where pooled RNA samples were required, samples were selected to assure acceptable ranges of expression levels for the seven cancer-related genes.

### RNA extraction

RNA extraction was performed using a semi-automated method, as previously described [8].

### RNA quantification

RNA was quantified using the RiboGreen fluorescence method (Invitrogen, Carlsbad, CA.), as previously described [9]. The minimum concentration required for accurate quantification was 5 ng/μl. Any sample with less than this amount was excluded from the study.

### Genomic DNA detection

RNA was tested for residual genomic DNA (gDNA) by quantitative PCR (qPCR) using an *ATCB *TaqMan^® ^assay (see section "TaqMan Primer Probe Design and Gene Expression Analysis" for details). Six wells containing 2 ng/well RNA were assayed per sample. RNA samples were failed if more than 2 wells gave a Cycle Threshold (C_T_) value of <37. This value represents less than 5 pg per well (less than 3 copies).

### Reverse transcription and qPCR

Reverse transcription was performed as a single, master reaction using the Omniscript kit (Qiagen, Valencia, CA) with a gene specific primer (one reverse primer) for each assay gene. Each primer in the RT reaction was at a concentration of 50 nmol/L. For the standard assay, a Tecan workstation was used to dispense RNA at a concentration of 2 ng/μl into the RT reaction (2 ng/well complementary DNA (cDNA) for quantitative PCR (qPCR)). The dilution series was manually pipetted using RNA input ranging from 0 to 32 ng/μl in the RT reaction (0 to 32 ng/well of cDNA for quantitative PCR). RT reactions were performed either in a single tube (dilution series) or 96-well plate (for standard reactions). The resulting cDNA was distributed to 384-well plates and PCR forward and reverse primers and TaqMan probe were added using a Tecan workstation.

### TaqMan^® ^primer probe design and gene expression analysis

Reference sequences were obtained from NCBI Entrez, and TaqMan assays designed using a proprietary primer-design module. Where possible, TaqMan assays were designed to span introns (*ATP5E, BGN, MK167, MYBL2, PGK1 *and *VDAC2*). Oligonucleotides were purchased from Integrated DNA Technologies (Coralville, IA). Dual labeled TaqMan probes have a Fluorescein **(**6-FAM™) 5' reporter and a Black Hole Quencher-2 (BHQ-2^®^) 3' quencher. Largest amplicon size was 84 base pairs and smallest size was 66 base pairs. See Additional file 1 Table S1 for the oligonucleotide sequences and Additional file 2 Table S2 for amplicon sequences.

TaqMan RT-PCR was performed using Applied Biosystems (ABI) Prism 7900HT instruments (7900) according to manufacturer's instructions. Reactions were 10 μl volume with cDNA equivalent to 2 ng total RNA (with the exception of the linearity study, where cDNA input ranged from 2^-10 ^to 2^5^ng/well). Final primer and probe concentrations were 0.9 μmol/L (primers) and 0.2 μmol/L (probe). PCR cycling conditions were 95°C for 10 minutes for one cycle, 95°C for 20 seconds, and 60°C for 45 seconds for 40 cycles. Triplicate wells were run for each assay and each well was classified as "valid" or "invalid" using pre-defined amplification curve metrics. The mean C_T _of the valid wells was used in subsequent statistical analyses.

### Reference gene normalization

Where required, normalization was performed using the average expression of the five reference genes (*ATP5E*, *GPX1*, *PGK1, UBB *and *VDAC2*). These genes were selected due to low expression variability between patients in the preliminary clinical development studies [6] (and data on file, Genomic Health Inc). The mean (valid) C_T _for each gene was subtracted from the mean (valid) C_T _for the 5 reference genes, then 10 was added to give a normalized range of expression from 0-15, where each unit reflects a 2-fold change in expression.

### Gene group and Recurrence Score calculation

Genes in the stromal gene group and cell cycle gene group were selected on the basis of co-expression and meaningful biological pathway associations, as previously described [6]. The CCGS was derived from the aggregated, normalized mean C_T _values for *MYC, MKI67 *and *MYBL2*. The SGS was derived from the aggregated, normalized mean C_T _values for *BGN, FAP *and *INHBA*. The CCGS, SGS and normalized mean C_T _for *GADD45B *are then entered into an algorithm to generate the RS (see Figure 1).

### Amplification efficiency

Using a pool of FPE RNA samples, two independent 17-point serial dilutions (representing 2^-10 ^to 2^5^ng RNA equivalent/well) were manually prepared and taken through RT and then qPCR, with triplicate wells per gene assay. The mean C_T _for the two dilution series, at each RNA input, was averaged to obtain a final non-normalized C_T _value. The estimates of amplification efficiencies were calculated for each of the 12 genes using the formula

$$\text{Efficiency}=2^{-1/\text{slope}}-1$$

where slope was estimated from the regression of C_T _measurements versus RNA concentration.

### Linearity

Using the aggregated, non-normalized C_T _values from the 17-point serial dilution, the linearity of C_T _value as a function of RNA concentration was evaluated for each gene assay. The polynomial method proposed by Krouwer et al., 1993 [10] and recommended by the National Committee of Clinical Laboratory Standards (NCCLS) [11], was used to test for linearity over the range of input RNA concentrations. The polynomial method evaluates nonlinearity in two parts. The first part examines whether a second or third order polynomial model fits better than a linear model. If none of the nonlinear terms in either the second or third order polynomial models is significant, then linearity is assumed. If significant nonlinearity is detected, then comparisons are made between the best-fitting polynomial model and linear polynomial to determine if the degree of bias is within a predefined allowable limit.

For each gene assay, orthogonal polynomial regression was used to obtain coefficients and associated tests of significance for the first (linear), second (quadratic), and third (cubic) order polynomials. Since precision is known to vary significantly across different RNA concentrations, heteroscedasticity in error variance was modeled through a log-linear variance model.

The degree of nonlinearity in signal response was assessed by examining the standard error of the regression and selecting the higher-order (nonlinear) polynomial model with the best fit. This statistic constituted the average difference between the data and the model, and so the model with the lowest value provided the best fit. At each (known) input RNA concentration, the deviation from linearity (DL) was calculated as follows:

$${\text{DL}}_{\text{i}}=p\left(x_{i}\right)-\left(b_{0}+b_{1}x_{i}\right)$$

where the values *x *range from *x_1..._x_15_*, and *p*(*x_i_*) was the value of the best-fitting polynomial at point *x_i_*. Consequently, DL_i _is a measure of the difference between the nonlinear model and the best-fit straight line at each of the RNA concentrations.

### Analytical sensitivity

The analytical sensitivity of each of the 12 genes was evaluated separately. Specifically, for each gene assay, a nonlinear mixed effects model with log-linear variance function was used to model the heteroscedasticity in intra-assay response as a function of RNA concentration. This information was used to estimate the Limit of Detection (LOD) and Limit of Quantitation (LOQ) of the assay. For each of the 12 genes, a lower one-sided 95% confidence interval on the C_T _at zero RNA concentration, y_min_, was calculated. The LOD was estimated by a lower 95% confidence bound on the mean expression at zero concentration (natural scale). Similarly, the LOQ was estimated by the inverse prediction of y_min _with the upper one-sided 95% confidence interval of the fitted calibration model. Analyses were performed using an iterative estimation scheme involving the PROC MIXED procedure in SAS version 9.1.

### Assay precision and reproducibility

Two FPE RNA pools were created to provide homogeneous templates for measuring precision and reproducibility; one pool had a RS in the high recurrence risk group (RS≥41) and the other had a RS in the low recurrence risk group (RS < 30). Using the standard 2 ng/well RNA equivalent, precision was assessed by estimating between-day, between-lot, between-7900 instrument (7900HT Fast Real-Time PCR System, Applied Biosystems), between-plate (within day), and within-plate variability components and total variability. Reproducibility was assessed by estimating differences in mean C_T_s between Tecan workstations used to assemble RT and qPCR plates. Analyses were performed using the PROC MIXED procedure in SAS version 9.1.

For assessing precision, a mixed effects analysis of variance (ANOVA) model was used to decompose the total variability in C_T _measurements into components of variance due to day, 7900 instrument, oligonucleotide lot and plate (run) within day, treating Tecan workstations as a fixed effect. An efficient (algorithmic) experimental design with G-efficiency > 50% was followed. Two Tecan workstations were used to assemble the RT and qPCR plates on five different days, using three different lots of oligonucleotides and data generated on five different 7900 instruments. For each gene, and each FPE RNA pool, Restricted Maximum Likelihood (REML) estimates of the precision of the assay were obtained along with approximate 95% confidence intervals expressed as relative standard deviations (RSD) of total variance.

For assessing the reproducibility of the assay, (least square) mean C_T _and mean scores between different combinations of RT and qPCR Tecan workstations were contrasted.

### Assay controls

Prior to clinical validation, a standard RNA template was run across several 7900 instruments, using various primer-probe lots to monitor control of the process. During clinical validation [7], the same RNA was included on every RT plate at the same concentration as test samples, and was used as an RT positive control. Nuclease-free water was used as the RT negative control. For the quantitative PCR controls, an RNaseP TaqMan primer-probe set (Applied Biosystems, Foster City, CA) was distributed in twelve wells across the 384-well plate; genomic DNA was added to the qPCR positive control wells and nuclease-free water to the qPCR negative control wells.

## Results

When an assay result depends on multiple single-value measurements, it is imperative that the analytical characteristics of each analyte are assessed independently. Here we describe the analytical performance of the individual components of the Onco*type *DX Colon Cancer Assay, which allowed us to confidently proceed to clinical validation.

### Amplification efficiency

Amplification efficiencies close to 100% are characteristic of qPCR assays that are robust, reproducible and specific. The 17-point serial dilution demonstrated that each gene assay achieved this. Specifically, amplification efficiencies (summarized in Table 1) were excellent and ranged from 96% to 107%. Pipette error, the presence of PCR inhibitors or non-specific amplification (primer-dimer) may account for efficiencies greater than 100%. In addition, all genes showed similar efficiencies. This is an important characteristic of tests which utilize expression of reference genes to normalize expression of the test genes [12].

**Table 1:** Amplification efficiency

Gene	Amplification Efficiency (%)	Lower 95% Confidence Interval	Upper 95% Confidence Interval
*ATP5E*	96.3	94.4	98.3

*BGN*	97.7	95.8	99.6

*MYC*	97.3	95.1	99.6

*FAP*	103.3	100.0	106.8

*GADD45B*	106.9	101.7	112.5

*GPX1*	97.8	95.5	100.2

*INHBA*	102.9	100.3	105.6

*MK167*	100.8	97.4	104.5

*MYBL2*	99.9	97.0	103.0

*PGK1*	97.0	95.3	98.7

*UBB*	98.0	95.4	100.6

*VDAC2.1*	97.1	95.3	98.9

Amplification efficiencies and 95% confidence intervals for the 12 genes. Amplification efficiencies range from 100% ± 7%, and vary due to additive assay error from the 17-point dilution series, PCR inhibition, or primer-dimer formation which can result in amplification efficiency greater than 100%.

### Linearity

The range of expression across patients for each of the 12 genes is large, and therefore it is important to demonstrate linearity of each gene assay over the potential range, to ensure accuracy of the result irrespective of the level of expression. Using the same dataset generated to assess amplification efficiency, the linearity of signal response between C_T _value and RNA concentration was evaluated for each of the 12 genes. Linear range estimates and estimated maximal deviation from linearity at the extremes are provided in Table 2. All 12 genes met the pre-specified acceptance criteria (a maximum deviation from linearity of 1 C_T_) over at least an 11 log_2 _concentration range (2^-6 ^to 2^5^) with a median deviation at the extremes of 0.4 C_T_.

**Table 2:** Linear range

Gene	Linear Range	Quadratic p-value	Cubic p-value	**Maximum Absolute Deviation from Linearity (C**_**T**_**)**
*ATP5E*	2 ^-10 ^to 2^5 ^ng	< 0.01	< 0.01	0.44

*BGN*	2 ^-9 ^to 2^5 ^ng	< 0.01	< 0.01	0.17

*MYC*	2 ^-10 ^to 2^5 ^ng	< 0.01	0.11	0.86

*FAP*	2 ^-7 ^to 2^5 ^ng	< 0.01	< 0.01	0.74

*GADD45B*	2 ^-7 ^to 2^5 ^ng	< 0.01	0.01	0.38

*GPX1*	2 ^-10 ^to 2^5 ^ng	< 0.01	0.04	0.30

*INHBA*	2 ^-7 ^to 2^5 ^ng	< 0.01	< 0.01	0.99

*MK167*	2 ^-7 ^to 2^5 ^ng	< 0.01	< 0.01	0.17

*MYBL2*	2 ^-6 ^to 2^5 ^ng	< 0.01	0.81	0.43

*PGK1*	2 ^-8 ^to 2^5 ^ng	< 0.01	< 0.01	0.56

*UBB*	2 ^-10 ^to 2^5 ^ng	< 0.01	0.08	0.36

*VDAC2*	2 ^-10 ^to 2^5 ^ng	< 0.01	< 0.01	0.23

Using the aggregated, non-normalized C_T _values from the 17-point serial dilution, the linearity of C_T _value as a function of RNA concentration was evaluated for each gene. All genes demonstrated a maximum deviation from linearity less than 1 C_T _over at least an 11 log_2 _concentration range (2^-6 ^to 2^5^).

### Analytical sensitivity

Since background noise could impact signal, it is important to determine the LOD for each gene. Similarly, it is important to establish the LOQ for each gene assay to achieve confidence in the level of expression being reported. Table 3 summarizes the estimated Limit of Detection (LOD) and Limit of Quantitation (LOQ) for each of the 12 genes. The LOD for all 12 genes was 40 C_T_, thus meeting the pre-specified acceptance criterion of ≥37 C_T_. The LOQ for all 12 genes was greater than 36 C_T_, also meeting the pre-specified acceptance criterion of ≥35 C_T_. These values are similar to those obtained for the 21 genes in the Onco*type *DX Breast Cancer Assay [13].

**Table 3:** Limit of detection (LOD) and limit of quantitation (LOQ)

Gene	LOD (C_T_)	LOQ (C_T_)	Log_2 _RNA Input (ng) at the LOQ
*ATP5E*	40.0	36.6	-10

*BGN*	40.0	36.9	-9

*MYC*	40.0	39.6	-10

*FAP*	40.0	38.3	-7

*GADD45B*	40.0	39.2	-7

*GPX1*	40.0	37.3	-10

*INHBA*	40.0	36.5	-7

*MK167*	40.0	37.1	-7

*MYBL2*	40.0	37.5	-6

*PGK1*	40.0	37.4	-8

*UBB*	40.0	37.6	-10

*VDAC2*	40.0	39.1	-10

LOD and LOQ were calculated using a nonlinear mixed effects model with log-linear variance function from the 17-point serial dilution series. LOD for all 12 genes was 40 C_T_, thus meeting the pre-specified acceptance criterion of ≥37 C_T_. The LOQ for all 12 genes was greater than 35 C_T_, also meeting the pre-specified acceptance criterion of ≥35 C_T_.

### Analytical precision

Between-7900 instrument, between-primer-probe lot, and within-PCR plate components of variance accounted for greater than 80% of total variance in C_T _measurements. Between-instrument and between-primer-probe lot constituted the largest components of variance. Table 4 lists the estimates of total variance for non-normalized C_T _values for each of the two FPE RNA pools investigated. All genes met the pre-specified acceptance criterion of 10% on the total % RSD. Total means and total SD for the SGS, CCGS, GADD45B and RS are summarized in Table 5. In both the low RS and high RS FPE RNA pools, the highest SD was 0.04 (normalized/aggregate) C_T _units for the SGS, 0.05 (normalized/aggregate) C_T _units for the CCGS, 0.13 (normalized/aggregate) C_T _units for GADD45B and 1.38 RS units for RS. These results demonstrate that the analytical variability would be very unlikely to produce a clinically meaningful change in the risk of recurrence reported for each patient.

**Table 4:** Precision for non-normalized CT values

FPE RNA Pool	Gene	Total SD (C_T_)	Overall C_T_	Total % RSD (95% CI)
1	*ATP5E*	0.24	24.86	1

				(0.6, 3.3)

1	*BGN*	0.22	25.89	0.8

				(0.5, 1.8)

1	*FAP*	0.28	29.86	0.9

				(0.5, 3.0)

1	*GADD45B*	0.32	30.81	1.1

				(0.6, 3.6)

1	*GPX1*	0.22	25.7	0.8

				(0.5, 2.4)

1	*INHBA*	0.25	28.52	0.9

				(0.5, 2.7)

1	*MK167*	0.2	28.56	0.7

				(0.4, 1.9)

1	*MYBL2*	0.23	29.27	0.8

				(0.5, 1.8)

1	*PGK1*	0.28	27.61	1

				(0.6, 2.3)

1	*UBB*	0.19	25.68	0.7

				(0.5, 1.7)

1	*VDAC2*	0.25	27.68	0.9

				(0.6, 2.1)

1	*MYC*	0.26	27.62	0.9

				(0.6, 2.2)

2	*ATP5E*	0.23	25.87	0.9

				(0.5, 3.7)

2	*BGN*	0.2	26.66	0.8

				(0.5, 1.8)

2	*FAP*	0.25	30.28	0.8

				(0.5, 2.8)

2	*GADD45B*	0.3	30.6	1

				(0.5, 4.5)

2	*GPX1*	0.19	26.05	0.7

				(0.4, 2.8)

2	*INHBA*	0.23	29	0.8

				(0.4, 2.9)

2	*MK167*	0.19	29.63	0.6

				(0.4, 1.6)

2	*MYBL2*	0.23	30.77	0.8

				(0.4, 2.9)

2	*PGK1*	0.24	28.64	0.8

				(0.5, 2.8)

2	*UBB*	0.16	26.17	0.6

				(0.4, 1.6)

2	*VDAC2*	0.24	28.87	0.8

				(0.5, 2.2)

2	*MYC*	0.24	30.32	0.8

				(0.5, 2.5)

Two FPE RNA pools were created to provide homogeneous templates for measuring precision. FPE RNA pool 1 represents a sample with a RS in the low risk group (RS < 30) and FPE RNA pool 2 represents a sample with a RS in the high risk group (RS≥40). Using the standard 2 ng/well RNA equivalent, precision was assessed by estimating between-day, between-lot, between-7900 instrument, between-plate (within day), and within-plate variability components and total variability. Listed are the estimates of total variance for each gene and each of the two FPET pools using raw C_T _values. All genes met the pre-specified acceptance criterion of 10% on the total %RSD.

**Table 5:** Precision for RS and RS components

FPET Pool	Score	Mean Score	Total SD
1	RS	23.6	1.18

2	RS	43.6	1.38

1	SGS	8.2	0.03

2	SGS	8.5	0.04

1	CCGS	7.8	0.04

2	CCGS	6.9	0.05

1	GADD45B	5.5	0.13

2	GADD45B	6.6	0.11

Two FPE RNA pools were created to provide homogeneous templates for measuring precision. FPE RNA pool 1 represents a sample with a RS in the low risk group (RS < 30) and FPE RNA pool 2 represents a sample with a RS in the high risk group (RS≥40). Using the standard 2 ng/well RNA equivalent, precision was assessed by estimating between-day, between-lot, between-7900 instrument, between-plate (within day), and within-plate variability components and total variability. Listed are the estimates of total variance for CCGS (normalized, aggregate C_T _values), SGS (normalized, aggregate C_T _values), GADD45B (normalized, aggregate C_T _values) and RS for each of the two FPET pools.

### Reproducibility

Table 6 summarizes the largest pair-wise differences in (least square) mean non-normalized C_T _between Tecan workstations by gene for the two FPE RNA pools. Overall, the differences in mean C_T _between Tecan workstations across all 12 genes and the two FPE RNA pools were small, all ≤ 0.28 C_T_. All the genes in the 12-gene panel met the pre-specified acceptance criteria for reproducibility.

**Table 6:** Reproducibly

FPET Pool	Gene	Difference in Mean C_T_
1	*ATP5E*	0.17

1	*BGN*	0.21

1	*FAP*	0.25

1	*GADD45B*	0.14

1	*GPX1*	0.16

1	*INHBA*	0.18

1	*MK167*	0.21

1	*MYBL2*	0.23

1	*PGK1*	0.28

1	*UBB*	0.22

1	*VDAC2*	0.19

1	*MYC*	0.26

2	*ATP5E*	0.08

2	*BGN*	0.08

2	*FAP*	0.11

2	*GADD45B*	0.13

2	*GPX1*	0.09

2	*INHBA*	0.05

2	*MK167*	0.1

2	*MYBL2*	0.15

2	*PGK1*	0.09

2	*UBB*	0.11

2	*VDAC2*	0.11

2	*MYC*	0.02

FPE RNA pool 1 represents a sample with a RS in the low risk group (RS < 30) and FPE RNA pool 2 represents a sample with a RS in the high risk group (RS≥40). To determine reproducibility of the assay, (least square) mean C_T _and mean scores between different combinations of RT and qPCR Tecan workstations were contrasted. Shown is the largest pair-wise differences in (least square) mean non-normalized C_T _between Tecan workstations by gene for the two FPE RNA pools. Overall, the differences in mean C_T _between Tecan workstations across all 12 genes and the two FPE RNA pools were small, all ≤0.28 C_T_.

### Assay controls

**Figure 2:**
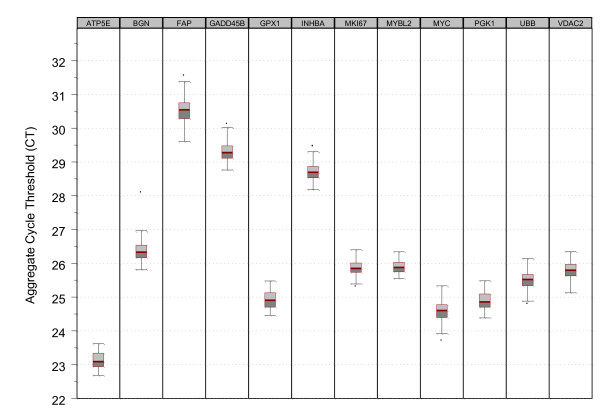
**Variability chart (box-plots) for RT positive controls run prior to and during clinical validation stratified by gene**. A standard RNA template was run across several 7900 instruments, using various primer-probe lots prior to and during clinical validation. The same RNA was included on every RT plate at the same concentration as test samples, and was used as an RT control. Nuclease-free water was used as the RT negative control. Standard deviations in aggregate cycle threshold measurements between samples ranged from 0.19 to 0.33, showing a highly controlled process.

**Figure 3:**
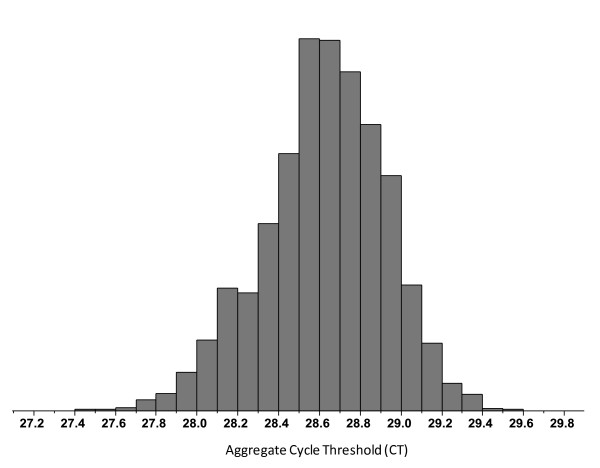
**Histogram of PCR positive controls (RNaseP) run prior to and during the clinical validation study**. An RNaseP TaqMan assay, with gDNA as the template, was distributed across twelve wells of a 384-well qPCR plate and used as the qPCR positive control. A pre-specified acceptance criterion of 1%CV was applied. During the clinical validation study, only twenty eight samples (2%) failed this 1% CV specification, demonstrating a highly controlled process.

A control sample was run on twenty-one RT plates prior to clinical validation of the Onco*type *DX Colon Cancer Assay, and on thirty one RT plates during clinical validation. Figure 2 is a variability chart (Box Plots) of gene expression for the 12 genes for the RT positive control run on these fifty two RT plates. It illustrates a tightly controlled process with standard deviations in aggregate C_T _measurements between samples ranging from 0.19 to 0.33 C_T _units. Figure 3 provides a histogram of the RNase P (Applied Biosystems. Foster City, CA) PCR positive controls from all PCR plates generated prior to and during clinical validation, where a pre-specified acceptance criterion of 1% coefficient of variance (CV) was applied. During the clinical validation study, seven PCR plates containing twenty eight samples (< 2%) failed this 1% CV specification and were therefore repeated.

## Discussion

A molecular diagnostic test used for treatment planning in cancer patients must be analytically robust and reproducible so as to enable consistent and accurate translation of each patient's tumor biology into clinically actionable information. The Onco*type *DX Colon Cancer Assay generates a RS for individual patients from the reference normalized tumor expression level of 7 cancer-related genes. These genes were chosen from a refined list of 48 genes (from an original set of 761 candidate genes) which were consistently associated with colon cancer recurrence in four independent clinical development studies comprising more than 1800 patients [6]. Among these 48 genes, two major pathways are represented; cell cycle and stroma-associated. The heart of the co-expressed stromal gene group is a collection of extra-cellular matrix (ECM) proteins including *BGN, COL1A1, SPARC *and *CTHRC1*. Cluster analysis from the development studies show these genes shared the lowest average distance between clusters (1-Pearson's R distance as low as 0.15) [6]. Other subgroups observed in the stroma-associated gene cluster included the TGFβ signaling pathway (*TGFBI, TGFB3, INHBA*), early response genes (*EGR1, GADD45B*), the WNT pathway (*SFRP2, SFRP4*), and invasion related genes (*PAI1, OPN, TIMP2, TIMP3*) [6]. Genes within the cell cycle group included checkpoint control genes and cell cycle regulation genes (such as *CDC20, MCM2, MYBL2, CSE1L, MYC *and *MK167*).

After identifying these biological pathways and genes as important markers of clinical outcome in stage II colon cancer, they were used to develop a test to enable translation of tumor biology into clinically useful information. Since biological heterogeneity was inherently embedded within the clinical validation of the Onco*type *DX Colon Cancer Assay, any such variability is already incorporated into the prediction of disease recurrence provided by each individual patient's RS. However, the most important source of variability from the perspective of daily testing is assay process, or analytical variability. The 12 RT-qPCR assays and associated processes were therefore analytically validated using pre-defined performance criteria prior to clinical validation of the RS. Analytical validation for the Onco*type *DX Colon Cancer Assay was patterned after the approach used for the widely accepted Onco*type *DX Breast Cancer Assay [3-5]. At the time the Onco*type *DX Breast Cancer Assay was analytically validated there was no widely accepted standard for multi-analyte RT-PCR tests. Therefore, methods commonly used to validate single-analyte laboratory tests were adapted for the purpose [13]. Here, all 12 individual genes were shown to be linear over a 2,000 fold range, and 5 genes (*ATP5E, MYC, GPX1, UBB *and *VDAC2*) were linear over a 32,000 fold range. For all genes, the limit of detection was at a C_T _of 40, and the limit of quantitation at a C_T _of 36 or greater, providing uniformly high analytical sensitivity for all genes being reported. The sensitivity and accuracy of the Onco*type *DX Colon Cancer Assay ensures robust reporting irrespective of the level of RNA expression, an attribute which may not be achieved with DNA microarrays given the lesser dynamic range of this platform [14,15]. This may in part account for why assessments and validations of microarray systems have focused on precision or reproducibility, rather than accuracy [16-20].

The Onco*type *DX Colon Cancer Assay is performed in a high-throughput process using multiple Tecan robotic workstations. In addition, multiple reagent lots and 7900 qPCR instruments provide potential sources of process variability. Therefore, all measureable analytical sources of variability were assessed to determine total system variability. Using two FPE RNA pools, an analysis of variance (ANOVA) model was applied to estimate the total analytical variability in C_T _measurements for each separate component of variance. The greatest source of analytical variability in C_T _(and therefore gene scores and RS) came from between-qPCR instruments and between-primer-probe lot components. However, even with these components the relative standard deviations (RSD) associated with each gene was still very small, and well within the pre-defined acceptance criterion of 10%. In fact, the upper bounds of the 95% confidence intervals on the RSD for all the genes in the 12-gene panel were within 10%. The high precision of the individual genes translates into a similarly high level of precision for the stromal gene group score (SD≤0.04), the cell cycle gene group scores (SD≤0.05) and the RS (SD≤1.38).

Plots from a control sample run prior to and during the clinical validation study demonstrate consistent stability for each gene assay. During the clinical validation study, less than 2% of the qPCR plates had to be repeated because of failure to meet both qPCR positive and negative control specifications. Such a well-controlled assay process is an important element of any prognostic or predictive molecular test performed in a clinical reference laboratory used for treatment planning.

The two principal co-expressed gene groups in the Onco*type *DX Colon Cancer Assay have long been known as important in cancer progression. The expression levels of an ECM protein (*BGN*), a fibroblast specific integral membrane serine protease (*FAP*), and a *TGFβ *family member (*INHBA*) are aggregated in the colon RS algorithm to form the SGS, where higher expression is associated with a higher risk of recurrence. Dvorak first described cancers as wounds that do not heal [21], and it is now generally accepted that activated stroma represents a "wound healing response" that can promote tumor growth, cell migration, invasion and angiogenesis [22-27]. In the same way that tissue regeneration during wound healing involves a complex relationship between the host and the microenvironment, tumorgenesis is also dependant on extra-cellular interactions and signals from the stroma. High amounts of stroma have been associated with poor clinical outcome in patients with colon cancer [28,29], but as demonstrated by clinical validation of the Onco*type *DX Colon Cancer Assay, the level of activation and associated gene expression within the stroma is strongly associated with risk of recurrence. *GADD45B *is entered into the algorithm as an individual gene, although it tends to associate with the larger stromal group on cluster analysis [6]. Interestingly, *GADD45B *is believed to stimulate *BGN *expression [30].

The second co-expressed gene group that proved to be clinically informative as a component of the colon cancer RS was the cell cycle gene group. In contrast to the Onco*type *DX Breast Cancer Assay, where higher expression of cell cycle genes (*STK15, MYBL2, MK167 *and *CCNB1*) is associated with increased risk of recurrence [1,2], higher expression of the colon cell cycle genes (such as *CDC20, MCM2, MYBL2, CSE1L, MYC *and *MK167*), was found to correlate with a lower risk of recurrence [6]. This is consistent with other reported evidence that cell cycle gene expression correlates with a good prognosis in colon cancer [31-35]. Garrity et al. reported only a weak correlation in colon cancer between *MK167 *levels and S-phase (the standard measure of proliferation) [32], indicating that expression of this gene may not signify rapidly dividing tumors. Instead, increased expression of these cell cycle checkpoint and control genes may represent tightened control of various stages of the cell cycle in response to DNA damage or misalignment of chromosomes during mitosis. *APC *is mutated in 80% of sporadic colon carcinomas [36] by either allelic loss or mutations in the multi cluster region (MCR), and there appears to be an interdependence of the two hits [37]. Homozygous deletions of *APC *are very rare, and residual APC activity is associated with difference biological characteristics depending on the type of mutation and its associated truncated protein (N-APC) [38-41]. For example, different *APC *mutations have been shown to result in various levels of β-catenin activation [38,39,42,43] and thus different growth advantages [40]. APC is also involved in chromosomal segregation, whereby it localizes to the ends of microtubules within the kinetochore and forms a complex with checkpoint proteins [35,44]. Some N-APC mutants have been shown to impair spindle checkpoint and contribute to mis-segregation of chromosomes [41,45-47]. With tighter cell cycle control and the ability (albeit a modest one) to undergo apoptosis or mitotic catastrophe in response to such mitotic errors, a tumor could reduce its abundance of aneuploidy and chromosomal instability (CIN) [48]. Given that CIN is associated with poor outcome [49-52] it could explain how high expression of cell cycle and checkpoint genes (such as *CDC20, MCM2, MYBL2, CSE1L, MYC *and *MK167*) were correlated with a lower risk of recurrence [6]. Since *APC *mutations are much rarer in breast cancer, it is likely that cell cycle genes (and specifically cell cycle control genes) do not harbor the same prognostic information as they do for colon cancer. This possible connection in colon cancer between different types of *APC *mutations, cell cycle control gene expression, CIN and prognosis warrants further investigation.

## Conclusions

In summary, after molecular markers and biological pathways had been identified in four independent clinical studies, the Onco*type *DX Colon Cancer Assay was developed to translate individual tumor biology into treatment planning. By requiring the individual components of RS to meet stringent analytical performance criteria, it was possible to clinically validate the Onco*type *DX Colon Cancer Assay and have confidence that every test performed in the clinical reference laboratory is done so using a sound and well controlled process.

## Competing interests

All authors are, or have been (in the last 5 years), employees of Genomic Health Inc. and hold shares and/or stock. Patents relating to the content of this manuscript have been applied for by Genomic Health Inc. Genomic Health Inc. funded this project.

## Authors' contributions

KMCL led the study and drafted the manuscript. CS and DW were responsible for study design and statistical analysis. JK coordinated and performed the analytical procedures. All authors read and approved the manuscript.

## Pre-publication history

The pre-publication history for this paper can be accessed here:

http://www.biomedcentral.com/1471-2407/10/691/prepub

## Supplementary Material

Additional file 1 **Oligonucleotide sequences**. Listed are the oligonucleotide sequences for all primers and probes. For each gene, the oligonucleotide of a forward primer, reverse primer and probe is provided. Click here for file

Additional file 2 **Amplicon sequences**. Listed are the amplicon length and sequence for each gene assay. Click here for file
